# Effectiveness and cost-effectiveness of an in-home respite care program in supporting informal caregivers of people with dementia: design of a comparative study

**DOI:** 10.1186/s12877-016-0373-4

**Published:** 2016-12-02

**Authors:** Sophie Vandepitte, Nele Van Den Noortgate, Koen Putman, Sofie Verhaeghe, Lieven Annemans

**Affiliations:** 1 Department of Public Health, Universiteit Gent, De Pintelaan 185, 9000 Ghent, Belgium; 2 Department of Internal Medicine, Universiteit Gent, De Pintelaan 185, 9000 Ghent, Belgium; 3 Department of Medical Sociology, Vrije Universiteit Brussel, Laarbeeklaan 103, 1090 Brussels, Belgium

**Keywords:** Informal caregivers, Effectiveness, Cost effectiveness, In-home respite care, Community-based respite care, Dementia, Comparative study

## Abstract

**Background:**

Frequent hospitalization and permanent nursing home placement not only affect the well-being of persons with dementia, but also place great financial strain on society. Therefore, it is important to create effective strategies to support informal caregivers so that they can continue to perform their demanding role. Preliminary qualitative evidence suggests that community-based respite services can actually be important for caregivers, and that the level of evidence should be further established in terms of effectiveness. Therefore, a comparative study to assess the effectiveness and cost-effectiveness of an in-home respite care program will be initiated.

**Methods:**

This manuscript described a quasi-experimental study to assess (cost)-effectiveness of an in-home respite care program to support informal caregivers of persons with dementia. Study population: 124 informal caregivers and persons with dementia will be included in the intervention group and will receive an in-home respite care program by an organization called Baluchon Alzheimer. 248 dyads will be included in the control group and will receive standard dementia care. The primary outcome is caregiver burden. Secondary outcomes are: quality of life of caregivers, frequency of behavioral problems of persons with dementia and the reactions of caregivers to those problems, intention to institutionalize the care-recipient, time to nursing home placement, resource use of the care-recipient, and willingness to pay for in-home respite care. When the trial demonstrates a difference in outcomes between both groups, within-trial and modeled cost-effectiveness analyses will be conducted in a separate economic evaluation plan to evaluate possible cost-effectiveness of the in-home respite care program compared to the control group receiving standard dementia care. Finally, the model based cost-effectiveness analyses will allow to extrapolate effects over a longer time horizon than the duration of the trial.

**Discussion:**

This study will have great added value because to date no studies measured effectiveness and cost-effectiveness of an in-home respite care program of the Baluchon type. Results of this trial can thus give much more insight in potential benefits and disadvantages of community-based respite care. Conclusions based on this trial can help policy-makers in elaborating future directions of dementia care.

**Trial registration:**

Clinicaltrials.gov Identifier NCT02630446.

## Background

Dementia is a known major public health problem with serious physical and emotional consequences for patients and their caregivers, and a high financial strain upon society. Knowing that on the one hand staying in the community may result in more quality of life for the patient and on the other hand there is a high cost of the disease for the society due to frequent hospitalization and permanent institutionalization in nursing homes, it can be stated that informal caregivers are essential in the care process and probably cost saving for society [1–7].

Although caregiving for a loved one can be very satisfying, it also demands a lot from informal caregivers over long periods due to the progressive decline of the disease [5, 8]. Most research of the past decade has shown that informal caregivers have more health problems, visit healthcare professionals more frequently, suffer from isolation, and have an increased risk of depression, distress and other illness [1, 2, 6, 8–12]. To prevent caregivers from getting overburdened, different supportive interventions, such as psychoeducation have been developed to improve their well-being.

Based on a recently conducted systematic review of comparative studies (randomized controlled trials and quasi-experimental studies), it was concluded that supporting caregivers is an effective strategy in improving well-being of caregivers and their recipients resulting in additional benefits for society. It also appeared that considerable research was done in investigating effectiveness of psychoeducational interventions, cognitive behavioral therapy, and occupational therapy, but research on the comparative effectiveness of respite care - which can be defined as a supportive service provided in or outside the home to give the informal caregiver a temporary relieve or beak from caregiving duties - was very rare [13].

An additional review, that also allowed observational designs without control group, was conducted to investigate the effectiveness of different types of respite care. In this review it was concluded that day care services are effective in decreasing caregiver burden and behavioral problems in people with dementia, but also accelerate time to nursing home placement. Results of temporary residential admission were rather mixed and showed unexpected adverse effects on both caregivers and care-recipients. No comparable evidence was found for night-time care in residential settings. Evidence on community-based respite care, including in-home respite care and host family respite care, is rare as well. Only one in-home respite care intervention was identified indicating some benefits for caregivers. Finally, there was no research on cost-effectiveness of any of the investigated types of respite care. As a result, the authors concluded that there is a need for new intervention studies measuring effectiveness and investigating cost-effectiveness of respite care. Especially the effect of an in-home respite program on the caregiver, the care-recipient, and the healthcare system should be further investigated [14].

On the other hand, substantial qualitative research indicated high satisfaction and positive perceptions regarding respite care in general and pointed out that breaks are crucial if caregivers are to continue to keep their loved ones home. Additionally, many caregivers pointed out their preference for in-home respite care, especially because in this way their loved ones can stay in their trusted environment [14].

Because current evidence suggests that respite services can actually be important and effective strategies for caregivers, but that the level of evidence should be further established in terms of effectiveness, especially for in-home respite care, we designed a quasi-experimental study to assess the effectiveness of an in-home respite care program compared to a control group not receiving the same type of in-home respite on the well-being of the caregiver, the care-recipient, and on the healthcare system. The latter in terms of resource use, intention to institutionalize the care-recipient, and time to nursing home placement.

When the trial demonstrates a difference in outcomes between both groups within-trial and modeled cost-effectiveness analyses will be conducted in a separate economic evaluation plan to evaluate possible cost-effectiveness of the in-home respite care program compared to the control group receiving standard dementia care. Finally, the model based cost-effectiveness analyses will allow to extrapolate effects over a longer time horizon than the duration of the trial.

The specific research questions to be answered are:

1. Is in-home respite care an effective strategy in supporting informal caregivers of people with dementia? Can in-home respite care decrease their burden, improve their reactions to behavioral problems and cause a difference in quality of life of the caregiver?
2. What impact does in-home respite care have on the frequency of behavioral problems of people with dementia?
3. What impact does in-home respite care have on the healthcare system in terms of resource use, intention to institutionalize, and time to nursing home admission?
4. Is in-home respite care a cost-effective strategy in supporting informal caregivers of people with dementia from the healthcare payer and societal perspective compared to no in-home respite care?
5. What are informal caregivers of persons with dementia willing to pay per day for in-home respite care?

## Methods/design

### Design

A quasi-experimental study is designed to assess the effectiveness of an in-home respite care program on caregivers of persons with dementia. The intervention group will consist of caregiver/care-recipient dyads receiving an in-home respite program called “Baluchonnage” and will be compared to a control group that does not receive this program. Comparison between the groups will be done by collecting health related and economic data.

Eventually, if the intervention is effective, modeled and trial based cost-effectiveness analyses will be undertaken in a separate economic evaluation plan. Analyses will be performed from the perspective of the healthcare payer (RIZIV/INAMI (National Institute for Health and Disability Insurance) and patient) and from a full societal viewpoint. Finally, the model based cost-effectiveness analyses will allow to project effects over a longer time horizon than the duration of the trial. An additional five-years’ time-horizon is chosen based on evidence of the average life expectancy in dementia [15, 16].

### Study participants

The study participants are caregiver/care-recipient dyads. The caregivers must be informal, meaning that they must not be professional healthcare workers in this caregiving role. They have to identify themselves as the main person responsible for the informal care (primary caregiver). Also, they must speak Dutch or French with some fluency and be able to read and write. Finally, caregivers will be excluded if they have cognitive impairments or severe psychiatric comorbidities. The care-recipient needs to be diagnosed with dementia and must live in the community. Evidence of symptoms will also be confirmed by testing for severity (Global Deterioration Scale) [17]. Finally, dyads of the control group must never had in-home respite of the Baluchon type, but be eligible and willing to receive it. On the other hand, dyads from the intervention group who have already received in-home respite care from the Baluchon type in the past are still allowed for inclusion in the intervention group.

### The in-home respite intervention

The in-home respite intervention group will receive in-home respite care also called “Baluchonnage” from Baluchon Alzheimer Belgium. Baluchon is a non-profit organization, founded in Québec by gerontologist Marie Gendron. She responded to the unmet need of many caregivers willing to take a break from caregiving duties, but lacking a solution for the person with dementia allowing them to stay home in their trusted environment. Eventually from this thought of mind, “Baluchonnage” was originated making it possible for those caregivers to take a short break while a trained support person (also called a “baluchonneuse” or “baluchonneur”) takes in their place. During this break, which can last up to two weeks, the caregiver has time to rest and return with new energy. For the person with dementia other resource use, structure, habits, and all daily activities remain the same. In this way Baluchon not only addresses to caregiver needs, but also to the need of the people with dementia feeling best in their trusted environment [7].

Twenty-four hours before the start of the respite period the “baluchonneur (se)” arrives at the home of the care-recipient. This transition period allows the “baluchonneur (se)” to get to know the environment, get to know the person with dementia and the caregiver, to build a trusting relationship, and to observe daily habits [7].

Additionally to the provision of respite, “Baluchonnage” also includes caregiver support by keeping a support diary during the respite period. In that diary the “baluchonneur (se)” writes down all daily experiences and strategies on how to manage the difficult behaviors the caregivers listed before. This support enables the caregiver to validate theirs perceptions, to learn how to deal with difficult behaviors and to feel understood by somebody. Even after the end of the respite period the “baluchonneur (se)” remains available for further counsel [7]. In Belgium this particular type of respite is not subsidized by the National Health Care Insurance.

Finally, based on ethical considerations, we cannot prohibit intervention participants from having multiple Baluchonnage-periods or utilize other standard dementia care services or supportive services including respite care. Especially not since some supportive respite care services such as: day care, night-time care (in-home or in a residential setting), and temporary residential admission are considered standard care in Belgium. Because use of other services or having multiple in-home respite periods via Baluchon can potentially influence study results, the extent of use will be captured with the RUD questionnaire (Resource Use in Dementia) [18].

### The control group - non in-home respite care

The control group receives usual dementia care. This means that they can have all types of standard dementia care (including medical, psychological, and other health and social services) and other supportive initiatives for informal caregivers including other types of respite care. As mentioned before, control group dyads who already utilized in-home respite from Baluchon, which is not considered standard dementia care in Belgium, will not be allowed for inclusion. Another reason for exclusion at baseline is not being eligible or willing to consider in-home respite via Baluchon.

### Sample size

Sample size calculation was performed using SPSS SamplePower 3®. First, literature was searched to determine the needed effect size and standard deviation (SD) allowing to distinguish a statistical significant and clinical relevant difference in the primary outcome, i.e. burden. Based on the findings of a similar high quality intervention study an effect size of 0.4 was used in the analysis, which implies a difference of six points on the ZBI scale (Zarit Burden Interview), and a SD (standard deviation) of 15 [19]. Also, an average drop-out rate of 20% was taken into account, a power of 80% and a significance level at 0.05. Based on these values a total of 124 caregiver/care-recipient dyads will be needed for the intervention group.

To reduce selection-bias inherent to quasi-experimental studies an allocation ratio of 1:2 will be used to allow matching techniques. As a result 248 caregiver/care-recipient dyads will be needed in the control group.

### Recruitment

Participants of the intervention group will be recruited by Baluchon staff when asking for a first or a new period of in-home respite care. First, Baluchon staff will describe the study to the dyads and ask them about their preparedness to participate. When they give their verbal consent, contact information will be forwarded to the research team who will then contact them by phone. During this call a home visit will be planned to sign the informed consent and complete the baseline assessment. In return for their participation, participants will receive the respite care for free during five days.

Participants for the control group will be recruited by several general practitioners spread over the different regions in Belgium, by physicians affiliated to the participating Belgian memory clinics, by several Belgian expert centers dementia, and by a Belgian sickness fund. Similar to the recruitment of the intervention group, the physician will give some information about the study. When verbal consent to participate is given to the physician, caregivers will be contacted by phone by the research team to check eligibility. This will be done by listing different support strategies (including “Baluchonnage”) and ask the caregiver if they would be interested to receive this type of support. When caregivers appear to be eligible the research team will plan a home visit to sign the informed consent and complete the baseline assessment.

### Data collection

**Fig. 1:**
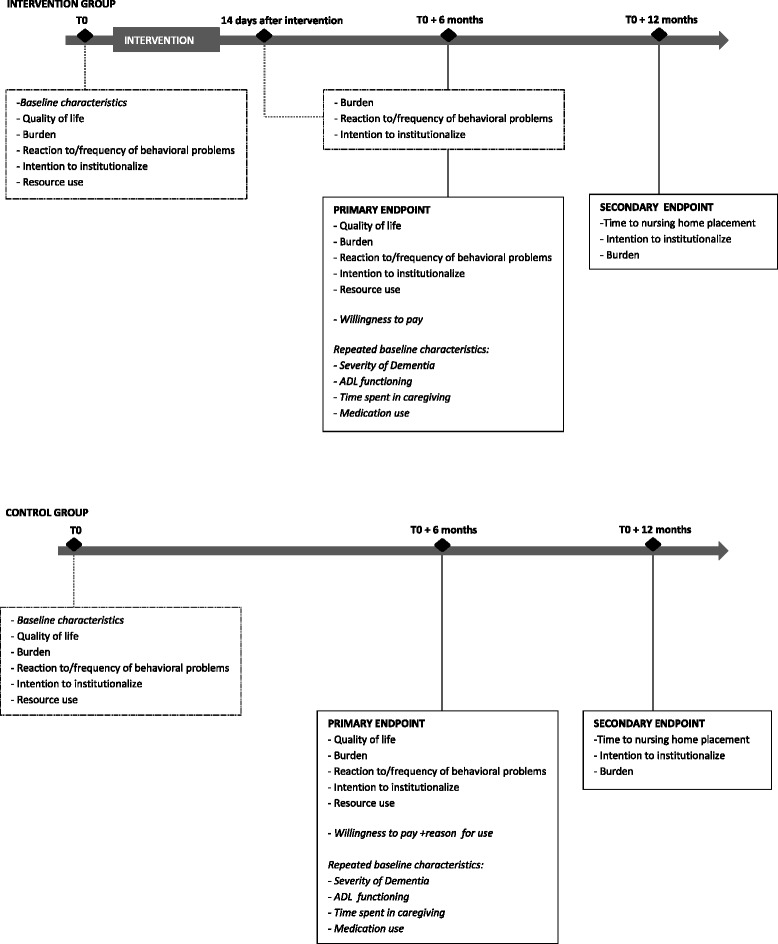
timeline of primary and secondary endpoints of the trial

Data collection of caregiver and care-recipient characteristics and research outcomes will be conducted at several assessment moments (Fig. 1). The baseline assessment will be conducted during a home visit by a member of the research team. Background characteristics and baseline values of the research outcomes will be gathered during this assessment. For the intervention group this will be in the week preceding the respite period. For the control group this will take place after inclusion. Two weeks after having the respite period possible changes in some of the study outcomes will be measured in the intervention group. Six months after inclusion, at the primary endpoint of this trial, all research outcomes (except time to nursing home placement) as well as some background characteristics (i.e. activities of daily living, dementia severity, dementia specific medication use & time spent in caregiving) will be measured again. Finally, 12 months after the baseline assessment, at secondary endpoint, some of the research outcomes will be measured again as well as time to nursing home placement.

To fulfill the assessments after the baseline assessment, caregivers will be contacted by telephone. This interviewing technique was chosen because it is the closest approach to face-to-face-interviewing which is not achievable in this trial due to time constraints and practical and organizational limitations. Permission to use the scales were asked to the authors or to the organizations responsible for the distribution of a certain questionnaire. All questionnaires are available in Dutch and French for use in Belgium. When no valid translation of a certain questionnaire existed in Dutch or French for Belgium a forward-back translation method was performed. In this method three phases were completed. First, a forward translation was done. This step was followed by a backward translation by another independent translator who had no knowledge or had no contact with the original questionnaire. During a last reconciliation phase, the original questionnaire was compared with the backward translation. In this way, discrepancies of translation were identified and corrected.

### Research outcomes and instruments

The trial will evaluate outcomes in the caregiver as well as in the care recipient (measured via the caregiver) and the healthcare system (Tables 1 and 2). The primary research outcome is caregiver burden. Secondary outcomes for caregivers are: health related quality of life and reactions to behavioral problems of the care-recipient. A secondary outcome related to the care-recipient is: frequency of behavioral problems. Secondary outcomes related to the healthcare system are: intention to institutionalize the recipient into a nursing home, and resource use of the recipient. Finally, in a follow up phase of the trial possible differences in time to nursing home placement will be measured (as well as burden and intention to institutionalize). Additionally, willingness to pay for “Baluchonnage” per day will be asked to the informal caregivers (Fig. 1, Tables 1 and 2).

**Table 1:** Data collection of the intervention group at the different assessment moments

Intervention group	Baseline (T0)	14 days after intervention (T1)	T0 + 6 months (T2)	T0 + 12 months (T3)
Background characteristics				
Age CG + CR	X			
Gender CG + CR	X			
Region CG + CR	X			
Etnicity CG + CR	X			
Marital status CG + CR	X			
Educational level CG + CR	X			
National Registration Number CR	X			
Relationship with CR	X			
Living situation of CG	X			
Employment status CG	X			
Waiting list nursing home	X			
Medication use Dementia-specific CR	X		X	
Earlier use of Baluchon	X			
Reason for respite	X			
Time spent in caregiving	X		X	
Severity of dementia CR	X		X	
ADL functioning CR	X		X	
Research outcomes				
Quality of life of CG	X		X	
Burden of CG	X	X	X	X
Frequency of behavioral problems of CR	X	X	X	
Reaction of CG on behavioral problems of CR	X	X	X	
Intention to institutionalize the CR	X	X	X	X
Resource use CR	X		X	
Willingness to pay			X	
Time to nursing home placement CR				X

*CG* Caregiver, *CR* care-recipient, *ADL* Activities of Daily Living

**Table 2:** Data collection of the control group at the different assessment moments

Control group	Baseline (T0)	14 days after intervention (T1)	T0 + 6 months (T2)	T0 + 12 months (T3)
Background characteristics				
Age CG + CR	X			
Gender CG + CR	X			
Region CG + CR	X			
Etnicity CG + CR	X			
Marital status CG + CR	X			
Educational level CG + CR	X			
National Registration Number CR	X			
Relationship with CR	X			
Living situation of CG	X			
Employment status CG	X			
Waiting list nursing home CR	X			
Medication use dementia-specific CR	X		X	
Earlier use of Baluchon	X			
Preparedness to receive in-home respite	X			
Reason for respite			X	
Time spent in caregiving	X		X	
Severity of dementia CR	X		X	
ADL functioning CR	X		X	
Research outcomes				
Quality of life of CG	X		X	
Burden of CG	X		X	X
Frequency of behavioral problems of CR	X		X	
Reaction of CG on behavioral problems of CR	X		X	
Intention to institutionalize the CR	X		X	X
Resource use CR	X		X	
Willingness to pay			X	
Time to nursing home placement CR				X

*CG* Caregiver, *CR* care-recipient, *ADL* Activities of Daily Living

#### Caregiver burden

Burden will be measured using the Zarit Burden Interview (ZBI) which is a 22-item validated self-report questionnaire developed to examine subjective burden of caregivers of people with dementia. The scale uses a five point Likert scale – ranged from never to nearly always present – to score every question. The final score is ranged between zero (low burden) to 88 (high burden). A validated Dutch and French version for use in Belgium is available [20, 21].

#### Health related quality of life of the caregiver

Health related quality of life will be measured using the EQ-5D-5 L. The EQ-5D-5 L is a valid extension of the 3-level questionnaire. It can be defined as a standardized non-disease specific value-based instrument to describe and value health related quality of life. The instrument consists of five health related domains (mobility, self-care, daily activities, pain, and depression/anxiety) to which a score from one to five can be given. Each score indicates the degree of perceived problems with the domain. Based on this score Quality Adjusted Life Years (QALYs) can be obtained for the cost-effectiveness analysis [22, 23]. Validated Dutch and French versions for Belgium are available as well as a valid telephone interviewing script in both languages.

#### Frequency of problematic behaviors and reactions of caregivers

To measure frequency of problematic behaviors in the care-recipient and the reaction of caregivers to these problems, the Revised Memory and Behavior Problems Checklist (RMBPC) will be used. This validated caregiver self-report measure contains 24 items including three domains (depression, memory-related problems and disruption) and two scales to be answered. One scale measures the frequency of problem behaviors of the recipient (five point scale from “never occurs” to “occurs daily or more often”) and the other measures the reactions of the caregiver to this behavior (five point scale form “not at all” to “extremely”). The described forward-back translation technique was used to translate the original English questionnaire into Dutch and French for Belgium [17].

#### Resource use and time spent in caregiving

Resource use of the care-recipient and also time spent in caregiving will be measured using the Utilization in Dementia instrument (RUD). This valid, standardized, and widely used instrument - available in Dutch and French for Belgium - can be used for collecting data on time spent in caregiving and resource use of dementia patients and their caregivers. To collect data on resource use of the person with dementia, caregivers only have to complete the part of the questionnaire measuring resource use of care-recipients [18].

Some small adjustments were made to measure resource use in order to better adapt the questionnaire to the Belgian context and to the setting of this trial. In contrast to the standard RUD questionnaire, amount of use of hospital admissions and care in a hospital emergency room will be asked for the last six months instead of over the last 30 days. Also, we added an additional table to the questionnaire containing the following respite services: day care (replaced from another table), night-time care (community-based or residential), short-stay, day sitting service, and in-home respite care (type Baluchon). For these respite services, potential use in the last six months will be asked. Additionally, a few Dutch words had to be changed to Dutch for Belgium [18].

Finally, information on potential use of other support will be collected separately by asking the caregivers if they currently receive any other supportive initiative for informal caregivers.

#### Intention to institutionalize the care-recipient into a long-term nursing home

The intention to institutionalize the care-recipient will be assessed using the Desire To Institutionalize scale (DTI) - originally developed by Morycz in 1985 [24] and slightly modified for use in the REACH II study [25]. Afterwards this modified version has also been validated by McCaskill et al. (2011) for use in different ethnical/racial groups [26]. This questionnaire contains six yes or no questions each measuring the caregiver’s desire to institutionalize the recipient into a nursing home, boarding home or assisted living. Because for this questionnaire no valid translations were available a forward-back translation method was used to translate the questionnaire in Dutch and French for Belgium.

#### Time to nursing home placement

Finally, the time to nursing home placement will be obtained by measuring the interval from the date of study enrollment to the date of permanent nursing home placement. Short term admissions in nursing home placements do not count as definitive nursing home placement. It is expected that even at 12 months a majority of patients will not be institutionalized. Hence, this parameter is to be considered as a secondary endpoint.

Additionally, the National Registration number of care-recipients will be obtained to allow us to trace study participants in the IMA database (Intermutualistisch Agentschap) and gather information on their date of placement in a nursing home over a longer time period than the trial.

#### Severity of dementia

Severity of dementia will be collected by using the Global Deterioration Scale (GDS) which is a valid and reliable instrument that distinguishes seven stages of dementia based on cognitive decline [27]. This questionnaire is not a neuropsychiatric test, but an instrument to objectively classify the severity of dementia. It can be administered by healthcare professionals and is often used in scientific research. A Dutch translation was available for the Netherlands. Two Belgian healthcare professionals were consulted to check appropriateness of this translation for the Belgian context. Also a French translation was made based on the original validated English version using the forward-back translation technique [28].

#### ADL functioning

Activities of Daily Living (ADL) of the care-recipient will be measured using a Belgian adapted version of the Katz Index of Independence in Activities of Daily Living [29]. The Belgian KATZ scale, which is an official instrument for mandatory use in Belgium, measures a person’s ability to perform activities of daily living based on a total score on six functions: bathing, dressing, toileting, transferring, continence, and feeding. Additionally, potential disorientation in time and place is asked in this questionnaire. In contrast to the original Katz index developed by Katz et al. [29] a score between one and four can be given for each domain of functioning. A score of four indicates total dependence (or total disorientation), while a score of one indicates total independence (no disorientation).

### Background characteristics

For caregivers we will obtain the following background characteristics: age, gender, region, marital status, ethnicity, educational level, relationship with the care-recipient, employment status, living situation, and time spent in caregiving. In both study groups the reason for (potential) use of respite will be asked based on evidence from the study of Connell et al. (2012) [30]. Earlier use of Baluchonnage will also be questioned to intervention group participants (Tables 1 and 2).

For the care-recipient the following background characteristics will be collected: age, gender, marital status, ethnicity, region, National Registration Number, educational level, being on a waiting list for long-term nursing home placement, dementia-specific medication use, activities of daily living (Belgian Katz scale), and severity of dementia (GDS). In a later phase, the National Registration Number of the person with dementia will allow us to trace study participants in the IMA database (Intermutualistisch Agentschap) and gather information on resource use and date of placement in a nursing home over a longer time period than the trial (Tables 1 and 2).

### Cost-effectiveness analysis

When appropriate, i.e. if the intervention demonstrates an effect in the study endpoints, within-trial and modeled cost-effectiveness analyses will be conducted in a separate economic evaluation plan [31].

First the analysis will be performed from the perspective of the healthcare payer taking into account direct healthcare costs for the government’s healthcare budget (RIZIV/INAMI) as well as patients’ co-payments [32]. Assuming that policy makers would consider reimbursement of in-home respite care of the Baluchon type in the future, the daily cost of Baluchonnage will be included in the analyses [33]. Alongside the costs of Baluchonnage other costs of healthcare resource use including hospital, residential, and community care will be included [31].

Additionally, also a full societal viewpoint will be undertaken not only including potential direct healthcare costs for the healthcare budget and patients, but also all other direct and indirect costs for caregivers and patients. Because in this viewpoint everyone affected by the intervention should be considered, caregiver time and costs (f.e. time spent in caregiving, productivity loss) as well as resource use and costs of other sectors (f.e. food delivery) should be included.

Based on the KCE guidelines (Belgian Health Care Knowledge Centre) for health economic evaluations in Belgium future costs will be discounted at 3% and future QALY’s at 1.5% [32]. Health utilities or QALY’s of caregivers from both intervention arms will be derived from the Belgian public preference list based on scores on the EQ-5D [34].

Decision analytic modeling will be carried out to extrapolate effects of the intervention found in the trial to a longer time horizon. The model will be based on the results from the trial as well as existing data from literature. Assumptions, hypotheses, and sources of information will be represented in a transparent and clear way. Finally, the model will be validated by experts.

The incremental cost-effectiveness ratio (ICER) will be calculated using the following equitation [34]:

$$ \begin{array}{l}\mathrm{ICER} = \underline {\Delta \mathrm{COSTS}}\left( difference\  between\  mean\  total\  cost\  of\  in tervention\  group\  and\  control\  group\right)\\ {}\kern4em \Delta \mathrm{QALYs}\left( difference\  between\  mean\  in\  QALY's\  of\  in tervention\  group\  and\  control\  group\right)\end{array} $$

### Resource use and costs

As already mentioned, data on resources used by the care-recipient of each study group will be obtained by an adapted version of the RUD instrument at baseline and after six months inclusion [18]. This RUD instrument attempts to include all resource use as defined by Drummond et al. [31] including: healthcare resource use containing hospital resources (f.e. in-patient and out-patient attendances), community care resources (f.e. general practitioner visits, nurse visits), caregiver and patient resources (f.e. time spent in caregiver), and resource use in other sectors (f.e. social worker visits, home help visits). Duration and frequency of the used services will be multiplied by each unit cost of the corresponding service. These unit costs will be obtained from the Belgian Reimbursement scheme using standard fees for regularly insured patients and other publicly available sources [18].

Time spent in caregiving will be calculated at baseline and after six months using the recall method for which also a part of the RUD instrument will be utilized. Next, the amount of time spent in caregiving will be monetized using the opportunity cost method which estimates the value of lost informal caregiver benefits due to spending time on providing informal care [35–37]. Productivity loss of the informal caregiver at productive age will not be taken into account because this would cause an overestimation due to double counting [31]. However, for caregivers at productive age we will gather information on whether their caregiving role effected their work situation.

During this trial, dyads of the intervention group will receive the intervention during five days for free. In normal circumstances patients must pay 65€ per day, while 350€ per day is covered by charity. However, to measure the total cost of the in-home respite care service under investigation in this trial, we will multiply the current unit cost of Baluchonnage per day (patient + charity) by the amount of days the in-home respite care was delivered. Additional costs above the fixed daily tariff - that patients must normally pay extra - will also be included such as: travel expenses during the respite period and having a pet (5€ extra per day) [33].

Additionally, willingness to pay for one day of in-home respite care by Baluchon will be obtained from both study groups using the contingent valuation method (CVM). This method can be defined as a stated preference method for eliciting a monetary value to a healthcare program. In this trial we use a closed response format [35, 36, 38–40].

Finally, for use in the decision analytic model, costs of nursing home placement will be derived from the average daily cost for staying in a nursing home in Belgium at the time of completing secondary endpoint.

### Within trial costs

Fixed intervention costs (f.e. program development, staff, and software) not changing based on the quantity of output, as well as variable costs (f.e. recruitment and assessment costs, intervention costs, and telephone and postage costs) varying on the quantity of output will be registered and then calculated into a total cost per caregiver/recipient dyad. Costs imposed by the study which are not part of routine practice will not be included in the cost analysis [31].

### Statistical analysis

First of all, descriptive statistics will be represented to draw a clear profile of the characteristics of study participants. Therefore the mean, percentages, and the standard deviations of quantitative variables will be displayed. To determine possible baseline differences between the groups, mean values of baseline characteristics will be compared using independent sample t-tests for continuous variables if normally distributed or by performing Pearson’s chi-square test for categorical variables. To help control for bias and confounding, statistical techniques such as propensity score matching will be used. Propensity score matching can be seen as a tool to simulate a RCT setting. In this way we can expect the observed effect to be an unbiased estimate of the real effect [41, 42].

To investigate possible effects of the intervention on the primary and secondary outcomes, analysis of variance will be conducted if the outcome variables are normally distributed. A *P*-value of 0.05 will be considered as significant. All analyses will be based on intention to treat also taking drop-outs into account and avoiding overestimation of respite care effects.

When the intervention is effective a cost-effectiveness analysis in a separate economic evaluation plan will be performed. ICERs will be calculated for the mean and upper and lower confidence levels of the costs and consequences. To explore uncertainty one-way-sensitivity analysis will be conducted around the ICER and illustrated in a Tornado diagram. Additionally, on all input variables a probabilistic sensitivity analysis, also called Monte Carlo analysis, will be conducted to test robustness of the model. These results will also be illustrated. Finally, the results and the willingness to pay threshold of the Belgian Health Care System will be presented in a cost-effectiveness acceptability curve [34].

To compare time to nursing home placement of persons who use respite care to those who do not, we will use Kaplan-Meier survival curves to illustrate association between the comparison groups. By additionally conducting a log-rank test statistical difference between the groups in time to placement can be found [43].

### Ethical issues

The study protocol has been approved by the Ethical Committee of the University Hospital of Ghent (B670201526906) prior to the beginning of the recruitment. Additionally, approval was obtained from the local Ethical Committees affiliated to the participating memory clinics. Written informed consent will be obtained from all patients before initiation. The study is registered at ClinicalTrials.gov: NCT02630446.

## Discussion

A first major strength of this trial is its contribution to the need for evidence on effectiveness and cost-effectiveness of in-home respite care. To date no other studies measured effectiveness and cost-effectiveness of an in-home respite service similar to the Baluchon service. Findings of this trial can also contribute to the elaboration of future directions of healthcare policies. After all, if in-home respite is effective in supporting informal caregivers and also cost-effective, policy makers should consider reimbursement of in-home respite care.

This trial has several methodological strengths often lacking in previous research. First of all, this study only uses international, validated, and widely used measurement tools to measure the research outcomes. Also, to better adapt some instruments to the Belgian context and this trial, adjustments were made for some instruments. When no valid translation in Dutch of French for Belgium was present we used a proper forward-back translation technique. Secondly, we performed a sample size calculation to know the necessary amount of caregivers needed to show an effect in the primary outcome given a certain effect size. In this analysis we also took into account an average drop-out rate to be expected. Additionally, intention-to-treat will be used to take into account drop-outs. To avoid bias and confounding, often occurring in non-randomized controlled trials, we will use propensity-score matching to simulate an RCT setting. In this way, the found intervention effects can be considered unbiased estimates of the real effect. Finally, active components of the intervention are clearly described, reasons for drop-out will be documented and characteristics of drop-out participants will be compared to those who completed the trial.

Despite the promising contribution of this study to community-based dementia care, several limitations need to be mentioned as well. First, there is a threat towards internal validity because the used quasi-experimental study design lacks random assignment of study groups. Although we use inclusion criteria to increase comparability and statistical efforts such as propensity score matching will be untaken, there can still be unmeasured confounders influencing causal inference. Nevertheless, for this particular research, a quasi-experimental design was preferable over a randomized controlled trial for feasibility and ethical reasons. First of all, the limited capacity of Baluchon Alzheimer Belgium makes it impossible to set up a RCT. Also, it can be seen as ethically unacceptable to randomly assign dyads to one of the trials arms. In this way some caregivers get free respite care while others cannot have this support during the trial.

Second, we only recruited caregivers asking for in-home respite care themselves in the intervention group and caregivers prepared to utilize this service in the control group. Therefore we cannot speculate whether effects might be different for caregivers not asking for or considering this type of support. Also, there is a high probability that caregiver/care-recipient dyads in the intervention group with lower socioeconomic status and lower educational level will be underrepresented because Baluchonnage is normally a rather expensive service that is not commonly known in Belgium. Nevertheless, we will use statistical techniques, such as propensity score matching, to respond to this problem. Also, the fact that participants receive the in-home respite care five days for free can also reduce the gap in socioeconomic status between intervention en control group.

Another important limitation of this trial is a consequence of not controlling the exact duration of the received in-home respite care period in the intervention group. The latter would not be acceptable due to ethical considerations and limited capacity of Baluchon Alzheimer Belgium. Even by setting a minimum duration of five days as an inclusion criterion, differences in outcome variables can still be caused by differences in duration. Nevertheless, to minimalize this phenomenon subgroup analyses can be undertaken. The same applies for intervention dyads who already received in-home respite in the past. After all, we can expect that dyads who are already familiar with in-home respite care react differently from people who are about to utilize it the first time. It is known that trust plays a crucial role in accepting respite care [44, 45]. Finally, use of other supportive strategies such as day care or night-time care can also influence results for which subgroup analyses can be undertaken if necessary.

Like other supportive interventions for caregivers this study only has a rather limited duration and a short follow up period. A longer time period could produce different outcomes or show certain effects to be strengthened, weakened, or sustained over time. Because dementia is a slow degenerative process, it can be expected that differences in time to nursing home placement between the study groups will not be found on such short notice. Therefore, to allow conclusions about possible difference in time to placement, we will measure intention to institutionalize which is seen as an adequate predictor for real placement into a nursing home [46]. Also, we will obtain the National Registration Number of dementia participants allowing to trace information on resource use and time to nursing home placement over a longer time period than the trial. Finally, we will perform modeled-based cost-effectiveness analyses to extrapolate results over a longer period.

Due to time constraints, organizational limitations, and high geographical distribution of study participants, assessments after the first baseline assessment will be done by telephone interviewing. Although no evidence was found indicating that the used instruments are not suited for telephone interviewing and data collection by telephone is widely performed and well accepted, face-to-face interviewing still remains the most preferred type of collecting data.

To measure differences in quality of life of caregivers, QALYs will be obtained from the EQ-5D. It is known that this generic preference-based measure of health lacks sensitivity in determining differences especially in emotional and mental health problems, and even more in short periods. Because it is to be expected from this intervention that in particular those emotional and mental health dimensions will shift, the question can be asked if the EQ-5D will be sensitive enough to show an effect on such short notice. Still, its use is preferred over the use of other disease-specific instruments, which have proven to be more sensitive, because the developed algorithms to translate the obtained scores into health utilities have been subject to a lot of criticism in literature.

Despite the described limitations, inherent to the complex nature of investigating respite care services, this study will have great added value because to date no studies measured effectiveness and cost-effectiveness of an in-home respite service similar to the Baluchon service. Results of this trial can give much more insight in potential benefits and disadvantages of community-based respite care, especially for informal caregivers and the healthcare system, but also for care-recipients. Conclusions based on this trial can thus help policy-makers in considering future directions of dementia care.

## Abbreviations

ADL: Activities of daily living
CVM: Contingent valuation method
DTI: Desire to institutionalize scale
EQ-5D: EuroQol 5 dimensions
GDS: Global deterioration scale
ICER: Incremental cost-effectiveness ratio
KCE: Belgian health care knowledge centre
QALY: Quality adjusted life years
RCT: Randomized controlled trial
RIZIV/INAMI: National Institute for Health and Disability Insurance
RMBPC: Revised memory and behavioral problems checklist
RUD: Resource use in dementia
SD: Standard deviation
ZBI: Zarit burden interview
